# Blood pressure increases are associated with weight gain and not antiretroviral regimen or kidney function: a secondary analysis from the ADVANCE trial in South Africa

**DOI:** 10.1002/jia2.26268

**Published:** 2024-07-08

**Authors:** Jennifer Manne‐Goehler, June Fabian, Simiso Sokhela, Godspower Akpomiemie, Nicholas Rahim, Samanta Tresha Lalla‐Edward, Alana T. Brennan, Mark J. Siedner, Andrew Hill, Willem Daniel Francois Venter

**Affiliations:** ^1^ Medical Practice Evaluation Center Massachusetts General Hospital Harvard Medical School Boston Massachusetts USA; ^2^ Division of Infectious Diseases Brigham and Women's Hospital Harvard Medical School Boston Massachusetts USA; ^3^ MRC/Wits Rural Health and Transitions Research Unit (Agincourt) University of the Witwatersrand Johannesburg South Africa; ^4^ Wits Donald Gordon Medical Centre School of Clinical Medicine Faculty of Health Sciences University of the Witwatersrand Johannesburg South Africa; ^5^ Wits Ezintsha, Faculty of Health Sciences University of the Witwatersrand Johannesburg South Africa; ^6^ Department of Global Health and Population Harvard T.H. Chan School of Public Health Boston Massachusetts USA; ^7^ Department of Global Health Boston University School of Public Health Boston Massachusetts USA; ^8^ Health Economics and Epidemiology Research Office University of the Witwatersrand Johannesburg South Africa; ^9^ Department of Epidemiology Boston University School of Public Health Boston Massachusetts USA; ^10^ Africa Health Research Institute KwaZulu‐Natal South Africa; ^11^ Massachusetts General Hospital Ringgold standard institution Boston Massachusetts USA; ^12^ University of Liverpool Liverpool UK; ^13^ Department of Public Health Medicine School of Health Systems and Public Health Faculty of Health Sciences University of Pretoria Pretoria South Africa

**Keywords:** hypertension, HIV, dolutegravir, tenofovir alafenamide, obesity, kidney function

## Abstract

**Introduction:**

Recent evidence has raised questions about whether newer HIV treatment regimens, including dolutegravir (DTG) and tenofovir alafenamide (TAF), are associated with increases in blood pressure (BP).

**Methods:**

We assessed changes in BP by treatment regimen and evaluated the relative contribution of kidney function and weight gain to these changes among participants in the ADVANCE phase‐3 trial clinical trial in South Africa (study dates: January 2017–February 2022). Our primary outcome of interest was a change in systolic BP (SBP) at 96 and 192 weeks, among those not receiving antihypertensive medication. The secondary outcome was treatment‐emergent hypertension at these same time points, defined as BP ≥140/90 mmHg on two occasions, or initiation of antihypertensive medication after week 4 among individuals without hypertension at enrolment. We used linear regression to evaluate the relationship between change in estimated glomerular filtration rate (eGFR) and change in SBP; and Poisson regression to evaluate the relationship between change in eGFR and treatment‐emergent hypertension at each time point. All models were adjusted for age, sex, treatment group and change in body mass index (BMI).

**Results:**

Over 96 weeks, the average changes in SBP were 1.7 mmHg (95% CI: 0.0−3.4), −0.5 mmHg (95% CI: −2.2 to 1.7) and −2.1 mmHg (95% CI: −3.8 to 0.4) in the TAF/emtricitabine (FTC)/DTG, tenofovir disoproxil fumarate (TDF)/FTC/DTG and TDF/FTC/efavirenz (EFV) groups, respectively. This difference was significant for the TAF/FTC/DTG compared to the TDF/FTC/EFV group (*p* = 0.002). Over 96 weeks, 18.2% (95% CI: 13.4–22.9), 15.4% (95% CI: 11.0–19.9) and 13.3% (95% CI: 8.9–17.6) of participants developed treatment‐emergent hypertension, respectively. In adjusted models, there was no significant relationship between change in eGFR and either outcome. Change in BMI was significantly associated with an increase in SBP, while age was associated with an increased risk of treatment‐emergent hypertension. Adjustment for BMI also mitigated the unadjusted relationship between HIV treatment regimen and SBP where present.

**Conclusions:**

In the ADVANCE cohort, weight gain and age accounted for increases in BP and risk of treatment‐emergent hypertension. HIV treatment programmes may need to integrate the management of obesity and hypertension into routine care.

**Clinical Trial Number:**

NCT03122262

## 1 INTRODUCTION

The evolution of antiretroviral therapy in the last decade has seen the introduction of integrase‐strand transfer inhibitors (INSTIs) in fixed‐dose combinations with tenofovir prodrugs, either tenofovir disoproxil fumarate (TDF) or tenofovir alafenamide (TAF), and nucleoside reverse transcriptase inhibitors, lamivudine or emtricitabine, as first‐line regimens across the globe [1, 2]. These combinations confer favourable side‐effect profiles, ease of dosing as once daily formulations and higher barriers to resistance over older regimens, and are recommended in most national guidelines [2, 3, 4, 5]. However, newer regimens have been associated with greater weight gain. Aside from a return‐to‐health effect, baseline factors shown to be associated with weight gain include lower CD4 cell count, higher HIV type 1 RNA, absence of injection drug use, female sex and black race—specifically in African populations and their diaspora [6]. The strongest associations with weight gain have been demonstrated in South African women, a demographic already vulnerable to the burgeoning epidemic of multimorbid non‐communicable disease [7]. Initial concerns that these antiretrovirals were causally linked to a rise in weight are now disputed, with weight changes probably due to a complex interplay between recovery from HIV‐induced inflammation and an obesogenic environment.

Recently, there have been concerns that these newer regimens may also be associated with a rise in blood pressure (BP), with several randomized and observational studies suggesting that INSTIs, specifically dolutegravir (DTG), and perhaps the nucleotide analogue TAF, may be associated with this rise [8, 9, 10, 11, 12]. However, the mechanism by which DTG or TAF may cause increases in BP remains unclear. Both drugs impact creatinine‐based measures of kidney function, estimated glomerular filtration rate (eGFR). However, while DTG alters tubular excretion of creatinine in urine causing a concomitant rise in serum creatinine concentrations, this is not associated with a true decline in kidney function, based on a more directly measured glomerular filtration rate. TAF is associated with a slight drop in eGFR, although why this would result in a BP change is also unclear [13]. INSTIs appear to exhibit different inflammatory profiles compared to other antiretroviral classes, which may affect arterial responses to BP [14, 15, 16]. Both drugs are associated with weight gain, which has also been linked to raised BP. Weight gain introduces additional measurement biases, as inappropriately small BP cuffs may result in artificially high readings in people with obesity [17]. Finally, the studies above may have multiple potential sources of bias, as several other studies have not shown an association with increased BP [18, 19, 20, 21].

To deepen the current understanding of the relationship between newer HIV treatment regimens and the risk of hypertension, we conducted a secondary analysis of a large randomized clinical trial of DTG with and without TAF compared to a previously widely used legacy regimen, where body weight, height and kidney function markers were collected, in an attempt to establish the relative contribution of each to increases in BP and treatment‐emergent hypertension.

## 2 METHODS

ADVANCE was a randomized, non‐inferiority phase‐3 trial done in Johannesburg, South Africa during which 1053 patients were enrolled from February 2017 through May 2018. Detailed methods and protocol, including sample size justification, are available, with the full 96‐ and 192‐week results also published [22, 23, 24]. In summary, participants aged 12 years or older with HIV and no antiretroviral exposure, and a creatinine clearance (Cockcroft–Gault formula) of more than 60 ml/minute (80 ml/minute if <19 years), received one of three regimens: coformulated TAF‐FTC (25–200 mg) and DTG (50 mg) as two tablets, or coformulated TDF‐FTC (300–200 mg) and DTG (50 mg) as two tablets, or coformulated TDF‐FTC and efavirenz (EFV, 600 mg) as one tablet, all administered daily. Participants were randomly assigned (1:1:1), and treatment allocation was not masked to staff or participants. All participants provided written informed consent and all study‐related procedures were conducted in accordance with the Declaration of Helsinki, and South African regulations. Study visits were done at weeks 4, 12, and then every 12 weeks, and included assessment of height, weight and BP, and laboratory investigations. Markers of kidney function other than creatinine were done through week 96. Concomitant medications, including the use of antihypertensive medications, were assessed at each study visit. Final study visits for the 192‐week results were completed in February 2022.

Height was measured once at baseline using the 2‐meter wall‐mounted metallic tape (Stature Meter). Weight was measured using a digital weight scale. BP was measured as a single reading with an automated machine (FORA Active Plus [p30 Plus] Blood Pressure Monitor, or Smart Gima Automatic Blood Pressure Monitor, or Rossman). However, only a standard cuff size was available. Part of the standard operating procedure each participant had a 5‐minute rest before each BP measurement. All instruments used for measuring vital signs were annually calibrated. Laboratory testing included serum creatinine, serum uric acid and urine albumin:creatinine ratio. Creatinine was measured with the Jaffe method and traceable to isotope dilution mass spectrometry. Urine Albumin was measured with the immunoturbidimetric assay, Tina‐quant Albumin Gen.2—Urine Application kit. Both assays were run on Cobas Integra 400 Plus.

### 2.1 Key outcome and exposure definitions

In this analysis, the primary outcomes of interest were continuous change in (1) systolic blood pressure (SBP) in mmHg and, separately (2) diastolic blood pressure (DBP) in mmHg. These outcomes were assessed at 96 weeks and again at 192 weeks. We considered these outcomes only among those participants who remained untreated for hypertension prior to randomization and over the respective time period; thus, participants who were being treated with antihypertensives at the time of screening or enrolment or who became treated with antihypertensives during the time horizon of the analysis (e.g. 96 or 192 weeks) were excluded.

The secondary outcome of interest was an integrated definition of “treatment‐emergent hypertension,” expressed in binary terms and defined by the following criteria: (1) a measured BP with SBP ≥ 140 mmHg and/or DBP ≥ 90 mmHg at a minimum of two different study visits between week 4 and the time horizon of the analysis (e.g. 96 or 192 weeks), or (2) the self‐reported initiation of treatment with an antihypertensive over that same time horizon. The 140/90 mmHg threshold was selected to be consistent with the South African Hypertension Society guidelines as a diagnostic threshold [25]. In this analysis, participants who (1) reported taking antihypertensive medication prior to or at the time of randomization or (2) had both a screening and enrolment BP with SBP ≥ 140 mmHg and/or DBP ≥ 90 mmHg were excluded from the analysis. Treatment‐emergent hypertension was further stratified into grade according to the Division of AIDS (DAIDS) Grading for the Severity of Adult and Pediatric Adverse Events [26]. The grade was defined by the highest BP measurement that contributed to the definition of treatment‐emergent hypertension. The DAIDS grading system for hypertension is defined as follows: Grade 1 = SBP <140 to 160 mmHg OR DBP 90 to <100 mmHg, Grade 2 = SBP ≥ 160 to < 180 mmHg OR DBP ≥100 to <110 mmHg, Grade 3 = SBP ≥180 mmHg OR DBP ≥ 110 mmHg, Grade 4 = life‐threatening consequences in a previously undiagnosed person or hospitalization required.

Our primary exposure of interest was the treatment regimen and considered as intention to treat based on randomization. For secondary exposures, we considered: (1) body mass index (BMI), which was derived from height and weight measures that were obtained at each visit and was calculated as weight in kilograms (kg) divided by height in meters squared (m^2^); change in BMI was calculated as the difference in BMI from screening to the stated time horizon (i.e. 96 or 192 weeks) of the analysis in kg/m^2^ and (2) kidney function, which was assessed using (i) creatinine‐based eGFR (ml/minute/1.73 m^2^) and calculated based on the race‐free Chronic Kidney Disease Epidemiology (CKD‐EPI) _(creatinine)_ 2021 equation; and (ii) urine albumin:creatinine ratio (uACR) (mg/mmol). Changes in eGFR and uACR were calculated from the screening visit to the time horizon of the analysis. Of note, the performance of the CKD‐EPI (creatinine) race‐free (2021) equation has been previously validated against iohexol‐measured GFR in South Africa; this prior research has demonstrated the limited accuracy of this equation for estimating GFR (P30 <80%) in black South Africans, though its use is still recommended in international guidelines and there are no better‐performing alternatives at present [27, 28, 29].

### 2.2 Statistical analysis

For the analysis of continuous BP change among participants who remained untreated for hypertension, we calculated the mean change in SBP and DBP, separately. These changes were calculated by study treatment assignment at 96 and 192 weeks. We assessed differences in means using two‐way tests of comparison for the TAF/3TC+DTG and TDF/FTC+DTG arms, each compared separately to the TDF/FTC/EFV arm and compared the means across all three groups using one‐way ANOVA. We then used linear regression to evaluate the relationship between change in eGFR and both change in SBP and, separately, change in DBP over 96 weeks and again at 192 weeks. These models were adjusted for age at enrolment (in years), sex, treatment regimen, baseline CD4 cell count and change in BMI.

To assess treatment‐emergent hypertension, we calculated the proportion of participants in each treatment group who had this secondary outcome at 96 and 192 weeks, respectively. We assessed differences in proportions using chi‐squared tests for the TAF/3TC+DTG and TDF/FTC+DTG arms, each compared to the TDF/3TC/EFV arm. We then used Poisson regression models with robust standard errors to evaluate the relationship between change in GFR and treatment‐emergent hypertension at 96 and 192 weeks. All models were adjusted for age (in years), sex, treatment regimen, baseline CD4 cell count (categorized as <100, 100−200, 200−300 and ≥300 cells/µl) and change in BMI. In sensitivity analyses, we also conducted all regression analyses stratified by sex. We then performed a survival analysis of time to hypertension diagnosis using a Cox modelling approach, adjusted for age (in years), sex, treatment regimen, baseline CD4 cell count, change in eGFR and BMI as a time‐varying covariate. Finally, we examined the correlation between SBP and uACR using Spearman correlation coefficients.

All analyses were conducted as complete case analyses; no missing data were imputed. These analyses were conducted in Stata v. 17.0.

### 2.3 Ethics approval

The ADVANCE study protocol was approved by the South African Health Products Regulatory Authority and local authorities, and the Medical Human Research Ethics Committee of the University of the Witwatersrand (Approval Certificate 160606B). The secondary analysis of these data was considered exempt by the Institutional Review Board of the Massachusetts General Brigham Hospital (#699).

## 3 RESULTS

The ADVANCE trial included 1053 participants, with 351 participants in each arm. The key demographic and health characteristics of the cohort have been published previously and are summarized in Table 1.

**Table 1:** Baseline characteristics of the ADVANCE cohort

Characteristic	Overall (*N* = 1053)	TAF/3TC/DTG (*N* = 351)	TDF/3TC/DTG (*N* = 351)	TDF/3TC/EFV (*N* = 351)	*p*‐value
***Age (mean [SD])***	32.5 (7.7)	32.5 (7.8)	32.4 (8.1)	32.4 (7.4)	0.965
***Sex (N [%])***					0.607
Female	623 (59.2)	214 (61)	208 (59.3)	201 (57.3)	
Male	430 (40.8)	137 (39)	143 (40.7)	150 (42.7)	
***BMI (mean [SD])***	24.1 (5.3)	24.1 (5)	24.1 (5.5)	24.1 (5.5)	0.996
***Weight, kg (mean [SD])***	68.8 (14.3)	68.7 (13.5)	68.6 (14.5)	69.2 (14.8)	0.845
***eGFR (mean [SD])***	115.9 (13.5)	116.8 (13.0)	115.6 (14)	115.5 (13.5)	0.334
***CD4 count per µl (N [%])***					0.280
≥ 300	514 (48.8)	186 (53)	154 (43.9)	174 (49.6)	
200−300	220 (20.9)	64 (18.2)	83 (23.6)	73 (20.8)	
100−200	196 (18.6)	59 (16.8)	69 (19.7)	68 (19.4)	
< 100	123 (11.7)	42 (12)	45 (12.8)	36 (10.3)	
***Systolic BP, mmHg (mean [SD])***	123 (15.4)	122 (14.7)	122.8 (15.3)	124.1 (16)	0.195
***Diastolic BP, mmHg (mean [SD])***	79.2 (11.9)	79.1 (11.6)	78.2 (11.5)	80.3 (12.6)	0.065
***uACR, mg/mmol (mean [SD])***	2.6 (11.4)	2.1 (6.6)	2.9 (12.6)	2.9 (13.7)	0.549

*Note*: Results presented as *N* (%) or mean (SD). *p*‐values estimated through either ANOVA or Chi‐squared test, where appropriate.

Abbreviations: BP, blood pressure; BMI, body mass index; CD4, clusters of differentiation 4; DTG, dolutegravir; eGFR, estimated glomerular filtration rate; FTC, emtricitabine; kg, kilogram; mg, milligram; mmHG, millimetres of mercury; mmol, millimole; SD, standard deviation; TAF, tenofovir alafenamide; TDF, tenofovir disoproxil fumarate; uACR, urine albumin‐creatinine ratio; µl, microlitre.

### 3.1 Primary outcome analysis

In the analysis of the primary outcome, we excluded 180 (17.1%) individuals who reported the use of an antihypertensive agent over the period from screening to 96 weeks. Additionally, another 187 (17.8%) participants were excluded due to missing BP measures at 96 weeks, leaving a sample of 686 in this analysis. Similarly, we excluded 200 (19.0%) individuals who reported the use of an antihypertensive agent over the period from screening to 192 weeks, along with an additional 411 (39.0%) participants due to missing BP measures at 192 weeks. This left a sample of 442 in this analysis. The demographic characteristics of the included sample overall and by treatment group are provided in the Supplementary Appendix (Tables S1−S6).

**Figure 1:**
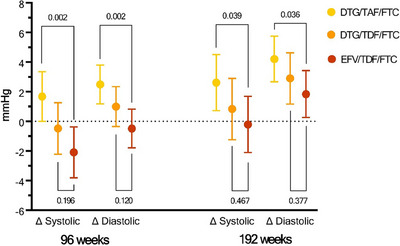
Changes in systolic and diastolic blood pressure from baseline to weeks 48 and 96, by ADVANCE treatment groups. Figure depicts estimates of the mean change in blood pressure measures, with error bars representing 95% confidence intervals. *p*‐values, listed above/below brackets, were estimated via two‐sample *t*‐tests. Sample restricted to those participants who did not report the use of hypertension medication at baseline or up to week 96 or 192 as applicable and had non‐missing measures of systolic or diastolic blood pressure. Abbreviations: DTG, dolutegravir; FTC, emtricitabine; mmHG, millimetres of mercury; TAF, tenofovir alafenamide; TDF, tenofovir disoproxil fumarate.

Over 96 weeks, the average change in SBP by treatment group was 1.7 mmHg (95% CI: 0.0−3.4), −0.5 mmHg (95% CI: −2.2 to 1.3) and −2.1 mmHg (95% CI: −3.8 to 0.4) in the TAF/FTC+DTG, TDF/FTC+DTG and TDF/FTC/EFV groups, respectively (Figure 1). In addition, we observed the following average changes in SBP by treatment group over 192 weeks: 3.6 mmHg (95% CI: −1.7 to 5.4), 0.7 mmHg (95% CI: 1.4−2.7) and −0.4 mmHg (95% CI: −2.2 to 1.4) in the TAF/FTC+DTG, TDF/FTC+DTG and TDF/FTC/EFV groups, respectively. Statistical comparison tests revealed that differences in SBP were only significant when comparing the TAF/FTC+DTG and the TDF/FTC/EFV groups at both 96 (*p* = 0.002) and 192 weeks (*p* = 0.039). In addition, three‐way comparisons of means via ANOVA showed *p* = 0.010 at 96 weeks and 0.127 at 192 weeks. The changes in DBP over 96 and 192 weeks followed a similar trend; their values and associated significance as compared to the TDF/FTC/EFV group are depicted in Figure 1 and provided in Supplementary Appendix (Tables S10 and S11).

In univariable linear regression models at both 96 and 192 weeks, the TAF/FTC+DTG treatment group and change in BMI were both significantly associated with greater SBP (Table 2). However, in multivariable‐adjusted models, the treatment group effect for TAF/FTC+DTG was substantially reduced at 96 weeks (β‐coefficient: 2.99 [0.28−5.70]) and was non‐significant at 192 weeks (β‐coefficient: 0.38 [−2.64 to 3.40]). Change in BMI remained significantly associated with SBP at both time points (β‐coefficients—96 weeks: 1.18 [0.81−1.55]); 192 weeks: 1.13 (0.73−1.53). There was no association between age, sex or change in eGFR, and SBP at either time point. The results for these regression analyses over 48 weeks and those considering DBP as the outcome are provided in Supplementary Appendix (Tables S13−S15).

**Table 2:** Linear regression models of the association of individual‐level characteristics and change in systolic blood pressure (mmHg) from baseline, adjusted and unadjusted

	Δ SBP at 96 weeks		Δ SBP at 192 weeks	
Characteristic	Unadjusted	Adjusted	Unadjusted	Adjusted
***Age***	−0.04 (−0.18 to 0.10)	−0.05 (−0.18 to 0.09)	0.07 (−0.09 to 0.23)	0.08 (−0.08 to 0.24)
***Sex***				
Female	1.96 (−0.06 to 3.98)	0.98 (−1.06 to 3.02)	1.54 (−0.73 to 3.82)	0.17 (−2.17 to 2.51)
***Group***				
TDF/FTC+DTG	1.61 (−0.81 to 4.04)	1.96 (−0.72 to 4.64)	1.04 (−1.76 to 3.85)	−0.11 (−3.12 to 2.91)
TAF/FTC+DTG	3.77 (1.33−6.20)	2.99 (0.28−5.70)	2.83 (0.04−5.61)	0.38 (−2.64 to 3.40)
***Change in BMI***	1.18 (0.81−1.55)	0.96 (0.54−1.37)	1.13 (0.73−1.53)	1.09 (0.64−1.54)
***Change in eGFR***	0.00 (−0.08 to 0.07)	0.08 (−0.01 to 0.16)	−0.06 (−0.14 to 0.01)	−0.02 (−0.10 to 0.07)
***CD4, count per µl***				
200–300	−1.03 (−3.56 to 1.50)	−1.35 (−3.86 to 1.16)	−1.13 (−4.03 to 1.76)	−1.87 (−4.73 to 1.00)
100−200	2.81 (0.15−5.48)	2.27 (−0.43 to 4.97)	1.72 (−1.31 to 4.75)	0.22 (−2.88 to 3.31)
< 100	5.41 (2.00−8.65)	3.68 (0.34−7.02)	1.66 (−2.14 to 5.46)	−0.11 (−3.95 to 3.73)

*Note*: Results are presented as point estimates (95% CI). Estimates derived from ordinary least‐squares linear regression models. Unadjusted models are univariable models in which change in systolic blood pressure is regressed on characteristic. Adjusted models are multivariable models in which change in systolic blood pressure is regressed on all of the included characteristics. “Change in BMI” is the difference between baseline BMI and BMI at week 96 and 192, respectively, measured in kg/m^2^. “Change in eGFR” is the difference between baseline eGFR and eGFR at week 96 and 192, respectively, measured in ml/minutes/1.73 m^2^. CD4 measured at baseline. Sample restricted to those participants who did not report the use of hypertension medication at baseline or up to week 96 or 192 when applicable and had non‐missing measures of systolic or diastolic blood pressure (96‐week *N* = 686; 192‐week *N* = 442).

Abbreviations: BMI, body mass index; CD4, clusters of differentiation 4; DTG, dolutegravir; eGFR, estimated glomerular filtration rate; FTC, emtricitabine; kg, kilogram; SD, standard deviation; TAF, tenofovir alafenamide; TDF, tenofovir disoproxil fumarate; µl, microlitre.

### 3.2 Secondary outcome analysis

Among all participants, 112 (10.6%) were (1) receiving antihypertensive treatment prior to or at the time of randomization or (2) had a BP of ≥140/90 mmHg at both screening and enrolment. Additionally, 189 (17.3%) and 429 (40.7%) participants were missing SBP measures at weeks 96 and 192, respectively. Once these individuals were excluded, total samples of 752 and 512 individuals remained in the 96‐ and 192‐week analyses of treatment‐emergent hypertension. Further characteristics of the sample included in this analysis overall and by treatment group are provided in the Supplementary Appendix (Tables S7−S9).

**Figure 2:**
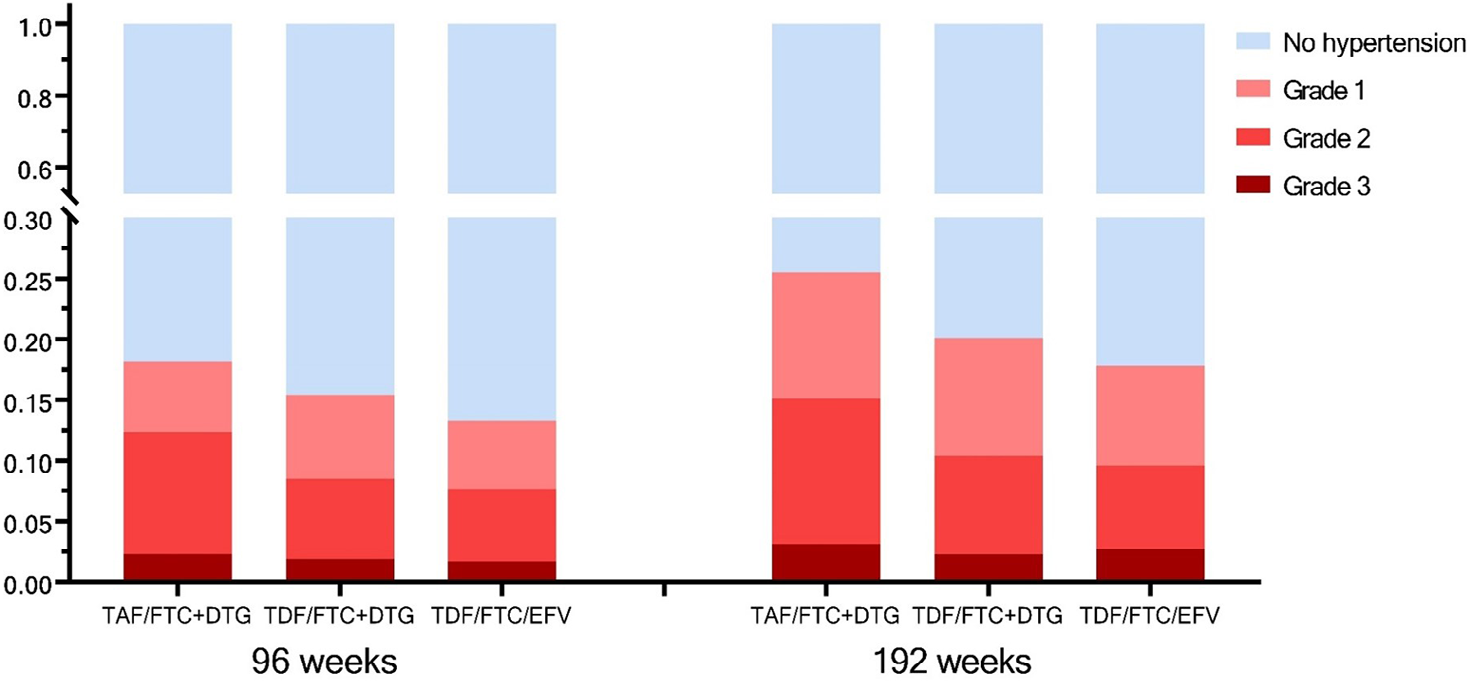
Proportion with treatment‐emergent hypertension and grade at weeks 96 and 192, by ADVANCE treatment group. Figure depicts estimates of the proportion of sample at each time point that have been deemed to have no hypertension, grade 1 hypertension, grade 2 hypertension and grade 3 hypertension by study standards. Sample restricted to those participants who did not report the use of hypertension medication at baseline or had two hypertensive blood pressure measures at baseline. Grade 1: SBP of 140−159 mmHg and/or DBP of 90−99 mmHg; Grade 2: SBP of 160−179 mmHg and/or DBP of 100−109 mmHg; Grade 3: SBP of ≥ 180 mmHg and/or DBP ≥ 110 mmHg; Grade 4: life‐threatening consequences or hospitalization. *p*‐values at 96 weeks: TAF/FTC+DTG versus TDF/FTC/EFV: *p* = 0.137; TDF/FTC+DTG versus TDF/FTC/EFV: *p* = 0.489; *p*‐values at 192 weeks: TAF/FTC+DTG versus TDF/FTC/EFV: *p* = 0.092; TDF/FTC+DTG versus TDF/FTC/EFV: *p* = 0.602. Abbreviations: DBP, diastolic blood pressure; DTG, dolutegravir; FTC, emtricitabine; mmHg, millimetres of mercury; SBP, systolic blood pressure; TAF, tenofovir alafenamide; TDF, tenofovir disoproxil fumarate.

Over 96 weeks, 18.2% (95% CI: 13.4−22.9), 15.4% (95% CI: 11.0−19.9) and 13.3% (95% CI: 8.9−17.6) of participants developed treatment‐emergent hypertension in the TAF/FTC+DTG, TDF/3TC/DTG and TDF/3TC/EFV groups, respectively (Figure 2). Over 192 weeks, 25.5% (95% CI: 19.3−31.7), 20.1% (95% CI: 14.1−26.1) and 17.8% (95% CI: 11.5−24.1) of participants developed treatment‐emergent hypertension in the TAF/FTC+DTG, TDF/FTC+DTG and TDF/FTC/EFV groups, respectively. These differences were non‐significant. Figure 2 depicts the composition of treatment‐emergent hypertension by grade. In regression analyses, age was associated with treatment‐emergent hypertension at 96 and 192 weeks in both unadjusted and adjusted models (RR: 1.07 [1.05−1.09]). There was no significant relationship between treatment group, change in eGFR or change in BMI and this outcome at either time point (Table 3). The proportion of people with treatment‐emergent hypertension and grade at weeks 48, 96 and 192 by treatment group are provided in Table S12.

**Table 3:** Poisson regression models of the association of individual‐level characteristics and risk of emergent‐hypertension during study period, adjusted and unadjusted

	Risk ratio at 96 weeks		Risk ratio at 192 weeks	
Characteristic	Unadjusted	Adjusted	Unadjusted	Adjusted
***Age***	1.07 (1.05−1.09)	1.07 (1.05−1.09)	1.07 (1.05−1.09)	1.07 (1.05−1.09)
***Sex***				
Female	0.78 (0.53−1.16)	0.85 (0.57−1.26)	0.81 (0.58−1.13)	0.85 (0.60−1.21)
***Group***				
TDF/FTC+DTG	1.64 (0.97−2.78)	1.83 (1.00−3.34)	1.17 (0.75−1.80)	1.25 (0.76−2.03)
TAF/FTC+DTG	1.55 (0.90−2.65)	1.70 (0.92−3.15)	1.37 (0.90−2.08)	1.37 (0.84−2.22)
***Change in BMI***	1.01 (0.92−1.10)	0.99 (0.89−1.10)	1.06 (0.99−1.13)	1.04 (0.97−1.12)
***Change in eGFR***	1.00 (0.98−1.01)	1.01 (0.99−1.02)	1.00 (0.99−1.01)	1.01 (0.99−1.02)
***CD4, count per µl***				
200−300	0.91 (0.53−1.55)	0.80 (0.48−1.35)	1.03 (0.65−1.62)	0.89 (0.57−1.39)
100−200	0.89 (0.51−1.55)	0.78 (0.45−1.37)	1.11 (0.70−1.77)	0.92 (0.58−1.46)
< 100	1.20 (0.67−2.15)	0.97 (0.52−1.78)	1.83 (1.19−2.84)	1.32 (0.82−2.13)

*Note*: Results are presented as risk ratios (95% CI). Estimates derived from Poisson regression models with robust standard errors. Unadjusted models are univariable models in which treatment‐emergent hypertension is regressed on characteristic. Adjusted models are multivariable models in which treatment‐emergent hypertension is regressed on all of the included characteristics. “Change in BMI” is the difference between baseline BMI and BMI at week 96 and 192, respectively, measured in kg/m^2^. “Change in eGFR” is the difference between baseline eGFR and eGFR at week 96 and 192, respectively, measured in ml/minutes/1.73 m^2^. CD4 measured at baseline. Sample restricted to those participants who did not report the use of hypertension medication at baseline or had two hypertensive blood pressure measures at baseline (96‐week *N* = 752, 192‐week *N* = 512).

Abbreviations: BMI, body mass index; CD4, clusters of differentiation 4; DTG, dolutegravir; eGFR, estimated glomerular filtration rate; FTC, emtricitabine; kg, kilogram; SD, standard deviation; TAF, tenofovir alafenamide; TDF, tenofovir disoproxil fumarate; µl, microlitre.

In the supplementary analysis, we found similar overall findings at 48 and 96 weeks (Table S16) and no major differences in sex‐stratified models (Tables S17 and S18). The relationship between individual characteristics and risk of baseline hypertension are displayed in Table S19 and the results of the Kaplan−Meier curves and Cox model of time to hypertension diagnosis are also displayed in Figure S1 and Table S20, respectively. Finally, there was no significant correlation between urine albumin:creatinine ratio and SBP (Table S21).

## 4 DISCUSSION

In this secondary analysis of the ADVANCE clinical trial, we found small increases in SBP among those receiving TAF/3TC+DTG who remained untreated for hypertension by 96 weeks; these increases were modest in absolute terms but were statistically significantly greater than for individuals receiving TDF/3TC/EFV. Moreover, the increases in SBP over the course of the study were associated with increases in body weight but in fully adjusted models, we found no significant relationship between changes in SBP and changes in kidney function. Adjustment for BMI also mitigated the unadjusted relationship between HIV treatment regimen and SBP where present. Furthermore, in these adjusted analyses, each unit of increase in BMI in kg/m^2^ was associated with an approximately 1 mmHg increase in BP. Importantly, there were high rates of treatment‐emergent hypertension by 96 weeks, with nearly one in five participants in the TAF/FTC+DTG arm and one in seven in the TDF/FTC+DTG arm developing this comorbidity, despite being a relatively young and otherwise healthy cohort at baseline.

Several observational studies have suggested that DTG or TAF may cause increases in BP. Among those analyses that have suggested this relationship, Brennan et al analysed a prospective cohort of people with HIV who were initiating antiretroviral therapy (ART) in Johannesburg from 2016 to 2020 [8]. This study found a 14.2% increase in the risk of hypertension, alongside greater weight gain, in those who initiated DTG compared to those who initiated EFV. In addition, Rivera et al conducted a retrospective propensity‐score matched analysis of adults in California who were initiating TAF versus TDF for HIV pre‐exposure prophylaxis [30] and found that TAF use was associated with a modestly increased risk of incident hypertension. Third, an analysis of participants from the RESPOND cohort concluded that INSTIs as a class were associated with a higher incidence of hypertension than non‐nucleoside reverse transcriptase inhibitor (NNRTIs), but rates were similar in those who used protease inhibitors [11].

In addition, there have also been secondary analyses of randomized controlled trials. These include a recent secondary analysis of the NEAT022 randomized clinical trial of immediate versus delayed transition to DTG. In this analysis, Sempere and colleagues did not find a significant difference in the incidence rate of hypertension, nor in SBP and DBP, among those who transitioned to DTG immediately compared to those who transitioned after a delay of 48 weeks [21]. Furthermore, our findings suggest that kidney function is an unlikely contributor to even small changes in BP, which instead appear to be linked more closely to weight gain. The NAMSAL trial found comparable rates of treatment‐emergent hypertension but greater differences in BP changes between arms. We hypothesize that differences between our analysis and the NAMSAL trial may have been due to higher rates of hypertension treatment in the ADVANCE cohort, differences in the extent of weight gain in South Africa compared to Cameroon and inclusion of a TAF‐containing arm in ADVANCE [31]. Ultimately, however, the lack of consensus in this literature suggests a need for additional research to clarify these relationships, including in other clinical trial populations and in other settings.

This study reinforces that hypertension and obesity should be considered key comorbidities in HIV medicine currently, including in southern Africa. Cardiovascular morbidity has rapidly replaced infectious diseases as a cause of death among people with HIV on treatment, who now have a near‐normal lifespan [32]. However, this comorbidity remains grossly under‐treated in all populations even in high‐income countries, despite exacting a large morbidity and mortality toll, with increasing calls for the integration of hypertension screening within HIV programmes [33, 34, 35]. The findings from this analysis are particularly critical for highlighting that, even when relatively young, people with HIV in South Africa are not only at risk of excess weight gain with the use of modern HIV treatment regimens but also are experiencing relatively high rates of treatment‐emergent hypertension. This supports the urgency of slowing South Africa's large and rising background epidemic of chronic non‐communicable diseases, including both obesity and hypertension, and emphasizes that this epidemiological transition may accelerate increases in cardiovascular disease risk as people age with treated HIV. Based upon our findings, it is likely that hypertension will become even more common, tracking with increases in body weight. To ensure the healthy ageing of people with HIV on modern ART, both obesity and hypertension service provision should be a focus of future HIV care programmes.

There are several limitations to this study. These include, first, the open‐label design, and differing pill burdens across arms. Second, consent processes and attrition may have introduced selection bias, in particular by the 192‐week visit. Of note, there was a slightly greater loss to follow‐up in the TDF/FTC/EFV group; this may have been due to the superior persistence and tolerability and lower risk of resistance in the DTG arms that made it more desirable to some study participants. However, the proportion of the original sample included in later analyses at 48 weeks did not differ significantly by arm.

Third, the study did not use standardized cuffs adjusted for arm sizes, as recommended in guidelines, or have a recommended standard operating procedure for the taking of BP by study staff [36]. Related to this is that the BP was measured only once at each time point. Fourth, there is no clear consensus about the optimal approach to analysing the risk of hypertension in this population; as such, we used two common approaches to define this construct, which yielded slightly different results in terms of factors associated with the risk of hypertension. Finally, the findings from this study may not be externally generalizable to other populations with HIV. Strengths include the prospective and randomized nature of the study, the inclusion of routine participants from HIV testing programmes and the high inclusion rates of women.

## 5 CONCLUSIONS

In summary, increases in BP and treatment‐emergent hypertension were common over 96 weeks in a young, healthy cohort of people with HIV initiating first‐line HIV treatment in South Africa and were associated with weight gain and age, respectively, rather than with antiretroviral regimen or kidney function changes. This suggests an urgent need for the integration of hypertension and obesity care into HIV services in this context.

## COMPETING INTERESTS

WDFV receives honoraria for educational talks and advisory board membership for Gilead, ViiV, Mylan/Viatris, Merck, Adcock‐Ingram, Aspen, Abbott, Roche, J&J, Sanofi, Boehringer Ingelheim and Virology Education. Wits Ezintsha receives funding from the Bill and Melinda Gates Foundation, SA Medical Research Council, National Institutes for Health, Unitaid, Foundation for Innovative New Diagnostics (FIND), Merck and the Children's Investment Fund Foundation (CIFF), has previously received funding from USAID and receives drug donations from ViiV Healthcare, Merck, J&J and Gilead Sciences for investigator‐led clinical studies.

## AUTHORS’ CONTRIBUTIONS

JM‐G, JF and WDFV conceived of the study, contributed to the initial and subsequent drafts, as well as to analysis.

## FUNDING

The ADVANCE study was funded by Unitaid, USAID, the South African Medical Research Council and ViiV Healthcare. JMG received funding from NIDDK K23125162. ATB received funding from NIDDK 1K01DK116929‐01A1.

## Supporting information


**Table S1**. Baseline characteristics of primary analytical sample at 48 weeks by treatment group.
**Table S2**. Baseline characteristics of primary analytical sample at 96 weeks by treatment group.
**Table S3**. Baseline characteristics of primary analytical sample at 192 weeks by treatment group.
**Table S4**. Baseline characteristics of primary analytical sample at 48 weeks.
**Table S5**. Baseline characteristics of primary analytical sample at 96 weeks.
**Table S6**. Baseline characteristics of primary analytical sample at 192 weeks.
**Table S7**. Baseline characteristics of secondary analytical sample at 48 weeks by treatment group.
**Table S8**. Baseline characteristics of secondary analytical sample at 96 weeks by treatment group.
**Table S9**. Baseline characteristics of secondary analytical sample at 192 weeks by treatment group.
**Table S10**. Changes in systolic and diastolic blood pressure (mmHg) from baseline to weeks 48 and 96, by ADVANCE treatment groups.
**Table S11**. Changes in systolic and diastolic blood pressure (mmHg) from baseline to week 192, by ADVANCE treatment groups.
**Table S12**. Proportion with treatment emergent hypertension and grade at weeks 48, 96, and 192, by ADVANCE treatment group.
**Table S13**. Linear regression models of the association of individual‐level characteristics and change in systolic blood pressure (mmHg) from baseline to week 48, adjusted and unadjusted.
**Table S14**. Linear regression models of the association of individual‐level characteristics and change in diastolic blood pressure (mmHg) from baseline, adjusted and unadjusted.
**Table S15**. Linear regression models of the association of individual‐level characteristics and change in diastolic blood pressure (mmHg) from baseline to week 48, adjusted and unadjusted.
**Table S16**. Poisson regression models of the association of individual‐level characteristics and risk of emergent‐hypertension up until week 48, adjusted and unadjusted.
**Table S17**. Sex‐stratified linear regression models of the association of individual‐level characteristics and change in systolic blood pressure (mmHg) from baseline to week 48, adjusted and unadjusted.
**Table S18**. Sex‐stratified Poisson regression models of the association of individual‐level characteristics and risk of emergent‐hypertension up until week 48, adjusted and unadjusted.
**Table S19**. Poisson regression models of the association of individual‐level characteristics and risk of baseline hypertension, adjusted and unadjusted.
**Table S20**. Adjusted Cox‐hazard model of time until hypertension diagnosis.
**Table S21**. Urine albumin:creatinine change and SBP change at 48 and 96 weeks by group.
**Figure S1**. Kaplan‐Meier curves of time until hypertension incidence, by treatment group.

## Data Availability

The data that support the findings of this study are available on request from the corresponding author. The data are not publicly available due to privacy or ethical restrictions.
