# Supplementary material for: Blood pressure increases are associated with weight gain and not antiretroviral regimen or kidney function: a secondary analysis from the ADVANCE trial in South Africa
Source: J Int AIDS Soc. 2024 Jul 8;27(7):e26268. doi: 10.1002/jia2.26268 (PMC11231445; doi:10.1002/jia2.26268)
Supplement: Supplementary file 1 — Table S1. Baseline characteristics of primary analytical sample at 48 weeks by treatment group. Table S2. Baseline characteristics of primary analytical sample at 96 weeks by treatment group. Table S3. Baseline characteristics of primary analytical sample at 192 weeks by treatment group. Table S4. Baseline characteristics of primary analytical sample at 48 weeks. Table S5. Baseline characteristics of primary analytical sample at 96 weeks. Table S6. Baseline characteristics of primary analytical sample at 192 weeks. Table S7. Baseline characteristics of secondary analytical sample at 48 weeks by treatment group. Table S8. Baseline characteristics of secondary analytical sample at 96 weeks by treatment group. Table S9. Baseline characteristics of secondary analytical sample at 192 weeks by treatment group. Table S10. Changes in systolic and diastolic blood pressure (mmHg) from baseline to weeks 48 and 96, by ADVANCE treatment groups. Table S11. Changes in systolic and diastolic blood pressure (mmHg) from baseline to week 192, by ADVANCE treatment groups. Table S12. Proportion with treatment emergent hypertension and grade at weeks 48, 96, and 192, by ADVANCE treatment group. Table S13. Linear regression models of the association of individual‐level characteristics and change in systolic blood pressure (mmHg) from baseline to week 48, adjusted and unadjusted. Table S14. Linear regression models of the association of individual‐level characteristics and change in diastolic blood pressure (mmHg) from baseline, adjusted and unadjusted. Table S15. Linear regression models of the association of individual‐level characteristics and change in diastolic blood pressure (mmHg) from baseline to week 48, adjusted and unadjusted. Table S16. Poisson regression models of the association of individual‐level characteristics and risk of emergent‐hypertension up until week 48, adjusted and unadjusted. Table S17. Sex‐stratified linear regression models of the association of individual‐lev [file JIA2-27-e26268-s001.docx]

**sTable 1. Baseline characteristics of primary analytical sample at 48 weeks by treatment group**

| **Characteristic** | **Overall**  **(N = 765)** | **TAF/FTC+DTG (N = 258)** | **TDF/FTC+DTG (N = 264)** | **TDF/FTC/EFV (N = 243)** | **p-value** |
| --- | --- | --- | --- | --- | --- |
| ***Age [mean (SD)]*** | 31.6 (7.3) | 31.5 (7.4) | 31.4 (7.2) | 32 (7.1) | 0.611 |
| ***Sex [N (%)]*** |  |  |  |  | 0.141 |
| Female | 456 (59.6) | 166 (64.3) | 148 (56.1) | 142 (58.4) |  |
| Male | 309 (40.4) | 92 (35.7) | 116 (43.9) | 101 (41.6) |  |
| ***BMI [mean (SD)]*** | 23.9 (5.2) | 23.9 (5.0) | 23.7 (5.0) | 24.0 (5.7) | 0.785 |
| ***Weight, kg [mean (SD)]*** | 68 (13.8) | 67.6 (13.4) | 67.7 (13.2) | 68.6 (14.9) | 0.682 |
| ***eGFR [mean (SD)]*** | 117.3 (12.6) | 118.2 (12.1) | 117.6 (12.1) | 116 (13.4) | 0.121 |
| ***CD4 count per uL [N (%)]*** |  |  |  |  | 0.389 |
| ≥ 300 | 368 (48.1) | 135 (52.3) | 112 (42.4) | 121 (49.8) |  |
| 200 - 300 | 161 (21) | 51 (19.8) | 59 (22.3) | 51 (21) |  |
| 100 - 200 | 145 (19) | 42 (16.3) | 59 (22.3) | 44 (18.1) |  |
| < 100 | 91 (11.9) | 30 (11.6) | 34 (12.9) | 27 (11.1) |  |
| ***Systolic BP, mmHg [mean (SD)]*** | 120 (12) | 118.7 (11.6) | 120.2 (12.4) | 121.1 (12) | 0.082 |
| ***Diastolic BP, mmHg [mean (SD)]*** | 76.8 (9.6) | 76.5 (9.3) | 76.3 (10) | 77.8 (9.5) | 0.177 |
| ***uACR, mg/mmol [mean (SD)]*** | 2.6 (10.9) | 2 (6.4) | 3.3 (14.3) | 2.5 (10.5) | 0.416 |

sTable 1 legend: Results presented as N (%) or mean (SD). P-values estimated through either ANOVA or Chi-squared test, where appropriate. Abbreviations: BP = blood pressure, BMI = body mass index, CD4 = clusters of differentiation 4, DTG = Dolutegravir, eGFR = estimated glomerular filtration rate, FTC = Emtricitabine, kg = kilogram, mg = milligram, mmHG = millimeters of mercury, mmol = millimole, SD = standard deviation, TAF = Tenofovir Alafenamide, TDF = Tenofovir Disoproxil Fumarate, uACR = urine albumin-creatinine ratio, uL = microliter.

**sTable 2. Baseline characteristics of primary analytical sample at 96 weeks by treatment group**

| **Characteristic** | **Overall**  **(N = 686)** | **TAF/FTC+DTG (N = 231)** | **TDF/FTC+DTG (N = 234)** | **TDF/FTC/EFV (N = 221)** | **p-value** |
| --- | --- | --- | --- | --- | --- |
| ***Age [mean (SD)]*** | 31.6 (7.3) | 31.3 (7.3) | 31.5 (7.3) | 32 (7.3) | 0.513 |
| ***Sex [N (%)]*** |  |  |  |  | 0.056 |
| Female | 410 (59.8) | 152 (65.8) | 129 (55.1) | 129 (58.4) |  |
| Male | 276 (40.2) | 79 (34.2) | 105 (44.9) | 92 (41.6) |  |
| ***BMI [mean (SD)]*** | 23.8 (5.2) | 23.9 (5.0) | 23.6 (5.1) | 23.9 (5.5) | 0.723 |
| ***Weight, kg [mean (SD)]*** | 67.7 (13.7) | 67.2 (13.2) | 67.4 (13.2) | 68.6 (14.7) | 0.514 |
| ***eGFR [mean (SD)]*** | 117.2 (12.5) | 118.1 (12.1) | 117.5 (12.2) | 115.8 (13.3) | 0.131 |
| ***CD4 count per uL [N (%)]*** |  |  |  |  | 0.448 |
| ≥ 300 | 326 (47.5) | 117 (50.6) | 97 (41.5) | 112 (50.7) |  |
| 200 - 300 | 151 (22) | 47 (20.3) | 57 (24.4) | 47 (21.3) |  |
| 100 - 200 | 131 (19.1) | 40 (17.3) | 52 (22.2) | 39 (17.6) |  |
| < 100 | 78 (11.4) | 27 (11.7) | 28 (12) | 23 (10.4) |  |
| ***Systolic BP, mmHg [mean (SD)]*** | 119.8 (11.7) | 118.4 (11.4) | 120.4 (12.2) | 120.7 (11.4) | 0.076 |
| ***Diastolic BP, mmHg [mean (SD)]*** | 76.7 (9.5) | 76.3 (9.1) | 76.4 (10.0) | 77.4 (9.3) | 0.376 |
| ***uACR, mg/mmol [mean (SD)]*** | 2.5 (10.3) | 2.2 (6.8) | 3.2 (15.0) | 1.9 (6.3) | 0.363 |

sTable 2 legend: Results presented as N (%) or mean (SD). P-values estimated through either ANOVA or Chi-squared test, where appropriate. Abbreviations: BP = blood pressure, BMI = body mass index, CD4 = clusters of differentiation 4, DTG = Dolutegravir, eGFR = estimated glomerular filtration rate, FTC = Emtricitabine, kg = kilogram, mg = milligram, mmHG = millimeters of mercury, mmol = millimole, SD = standard deviation, TAF = Tenofovir Alafenamide, TDF = Tenofovir Disoproxil Fumarate, uACR = urine albumin-creatinine ratio, uL = microliter.

**sTable 3. Baseline characteristics of primary analytical sample at 192 weeks by treatment group**

| **Characteristic** | **Overall**  **(N = 442)** | **TAF/FTC+DTG (N = 157)** | **TDF/FTC+DTG (N = 151)** | **TDF/FTC/EFV**  **(N = 134)** | **p-value** |
| --- | --- | --- | --- | --- | --- |
| ***Age [mean (SD)]*** | 32.5 (7) | 32.1 (6.6) | 32.6 (7.4) | 32.8 (6.9) | 0.644 |
| ***Sex [N (%)]*** |  |  |  |  | 0.061 |
| Female | 249 (56.3) | 100 (63.7) | 81 (53.6) | 68 (50.7) |  |
| Male | 193 (43.7) | 57 (36.3) | 70 (46.4) | 66 (49.3) |  |
| ***BMI [mean (SD)]*** | 23.9 (5.3) | 23.8 (4.8) | 23.8 (5.3) | 24.1 (5.8) | 0.874 |
| ***Weight, kg [mean (SD)]*** | 68.4 (13.9) | 67.7 (12.8) | 67.8 (13.4) | 69.8 (15.5) | 0.366 |
| ***eGFR [mean (SD)]*** | 115.4 (12.4) | 116 (11.9) | 116.3 (12.2) | 113.5 (13.1) | 0.119 |
| ***CD4 count per uL [N (%)]*** |  |  |  |  | 0.381 |
| ≥ 300 | 208 (47.1) | 81 (51.6) | 59 (39.1) | 68 (50.7) |  |
| 200 - 300 | 99 (22.4) | 31 (19.7) | 41 (27.2) | 27 (20.1) |  |
| 100 - 200 | 87 (19.7) | 30 (19.1) | 32 (21.2) | 25 (18.7) |  |
| < 100 | 48 (10.9) | 15 (9.6) | 19 (12.6) | 14 (10.4) |  |
| ***Systolic BP, mmHg [mean (SD)]*** | 120.7 (11.5) | 119.2 (10.2) | 121.9 (12.3) | 121.2 (11.9) | 0.100 |
| ***Diastolic BP, mmHg [mean (SD)]*** | 77.1 (9.2) | 76.6 (8.1) | 77 (9.9) | 77.8 (9.7) | 0.504 |
| ***uACR, mg/mmol [mean (SD)]*** | 2.1 (8.6) | 1.6 (3.6) | 2.7 (13.2) | 1.9 (6.0) | 0.494 |

sTable 3 legend: Results presented as N (%) or mean (SD). P-values estimated through either ANOVA or Chi-squared test, where appropriate. Abbreviations: BP = blood pressure, BMI = body mass index, CD4 = clusters of differentiation 4, DTG = Dolutegravir, eGFR = estimated glomerular filtration rate, FTC = Emtricitabine, kg = kilogram, mg = milligram, mmHG = millimeters of mercury, mmol = millimole, SD = standard deviation, TAF = Tenofovir Alafenamide, TDF = Tenofovir Disoproxil Fumarate, uACR = urine albumin-creatinine ratio, uL = microliter.

**sTable 4. Baseline characteristics of primary analytical sample at 48 weeks**

| **Characteristic** | **Overall**  **(N = 1053)** | **Excluded**  **(N = 288)** | **Included**  **(N = 765)** | **p-value** |
| --- | --- | --- | --- | --- |
| ***Age [mean (SD)]*** | 32.5 (7.7) | 34.6 (8.6) | 31.6 (7.3) | < 0.001 |
| ***Sex [N (%)]*** |  |  |  | 0.633 |
| Female | 623 (59.2) | 167 (58) | 456 (59.6) |  |
| Male | 430 (40.8) | 121 (42) | 309 (40.4) |  |
| ***BMI [mean (SD)]*** | 24.1 (5.3) | 24.7 (5.5) | 23.9 (5.2) | 0.018 |
| ***Weight, kg [mean (SD)]*** | 68.8 (14.3) | 71.1 (15.1) | 68 (13.8) | 0.001 |
| ***eGFR [mean (SD)]*** | 115.9 (13.5) | 112.3 (15.1) | 117.3 (12.6) | < 0.001 |
| ***CD4 count per uL [N (%)]*** |  |  |  | 0.896 |
| ≥ 300 | 514 (48.8) | 146 (50.7) | 368 (48.1) |  |
| 200 - 300 | 220 (20.9) | 59 (20.5) | 161 (21) |  |
| 100 - 200 | 196 (18.6) | 51 (17.7) | 145 (19) |  |
| < 100 | 123 (11.7) | 32 (11.1) | 91 (11.9) |  |
| ***Systolic BP, mmHg [mean (SD)]*** | 123 (15.4) | 130.9 (19.9) | 120 (12) | < 0.001 |
| ***Diastolic BP, mmHg [mean (SD)]*** | 79.2 (11.9) | 85.4 (14.9) | 76.8 (9.6) | < 0.001 |
| ***uACR, mg/mmol [mean (SD)]*** | 2.6 (11.4) | 2.7 (12.5) | 2.6 (10.9) | 0.945 |

sTable 2 legend: Results presented as N (%) or mean (SD). P-values estimated through either t-test or Chi-squared test, where appropriate. Abbreviations: BP = blood pressure, BMI = body mass index, CD4 = clusters of differentiation 4, DTG = Dolutegravir, eGFR = estimated glomerular filtration rate, FTC = Emtricitabine, kg = kilogram, mg = milligram, mmHG = millimeters of mercury, mmol = millimole, SD = standard deviation, TAF = Tenofovir Alafenamide, TDF = Tenofovir Disoproxil Fumarate, uACR = urine albumin-creatinine ratio, uL = microliter.

**sTable 5. Baseline characteristics of primary analytical sample at 96 weeks**

| **Characteristic** | **Overall**  **(N = 1053)** | **Excluded**  **(N = 367)** | **Included**  **(N = 686)** | **p-value** |
| --- | --- | --- | --- | --- |
| ***Age [mean (SD)]*** | 32.5 (7.7) | 34.0 (8.3) | 31.6 (7.3) | < 0.001 |
| ***Sex [N (%)]*** |  |  |  | 0.587 |
| Female | 623 (59.2) | 213 (58.0) | 410 (59.8) |  |
| Male | 430 (40.8) | 154 (42.0) | 276 (40.2) |  |
| ***BMI [mean (SD)]*** | 24.1 (5.3) | 24.7 (5.5) | 23.8 (5.2) | 0.006 |
| ***Weight, kg [mean (SD)]*** | 68.8 (14.3) | 70.8 (15.1) | 67.7 (13.7) | 0.001 |
| ***eGFR [mean (SD)]*** | 115.9 (13.5) | 113.6 (14.9) | 117.2 (12.5) | < 0.001 |
| ***CD4 count per uL [N (%)]*** |  |  |  | 0.520 |
| ≥ 300 | 514 (48.8) | 188 (51.2) | 326 (47.5) |  |
| 200 - 300 | 220 (20.9) | 69 (18.8) | 151 (22) |  |
| 100 - 200 | 196 (18.6) | 65 (17.7) | 131 (19.1) |  |
| < 100 | 123 (11.7) | 45 (12.3) | 78 (11.4) |  |
| ***Systolic BP, mmHg [mean (SD)]*** | 123 (15.4) | 128.9 (19.2) | 119.8 (11.7) | < 0.001 |
| ***Diastolic BP, mmHg [mean (SD)]*** | 79.2 (11.9) | 83.9 (14.4) | 76.7 (9.5) | < 0.001 |
| ***uACR, mg/mmol [mean (SD)]*** | 2.6 (11.4) | 3 (13.3) | 2.5 (10.3) | 0.492 |

sTable 5 legend: Results presented as N (%) or mean (SD). P-values estimated through either t-test or Chi-squared test, where appropriate. Abbreviations: BP = blood pressure, BMI = body mass index, CD4 = clusters of differentiation 4, DTG = Dolutegravir, eGFR = estimated glomerular filtration rate, FTC = Emtricitabine, kg = kilogram, mg = milligram, mmHG = millimeters of mercury, mmol = millimole, SD = standard deviation, TAF = Tenofovir Alafenamide, TDF = Tenofovir Disoproxil Fumarate, uACR = urine albumin-creatinine ratio, uL = microliter.

**sTable 6. Baseline characteristics of primary analytical sample at 192 weeks**

| **Characteristic** | **Overall**  **(N = 1053)** | **Excluded**  **(N = 611)** | **Included**  **(N = 442)** | **p-value** |
| --- | --- | --- | --- | --- |
| ***Age [mean (SD)]*** | 32.5 (7.7) | 32.4 (8.3) | 32.5 (7) | 0.923 |
| ***Sex [N (%)]*** |  |  |  | 0.112 |
| Female | 623 (59.2) | 374 (61.2) | 249 (56.3) |  |
| Male | 430 (40.8) | 237 (38.8) | 193 (43.7) |  |
| ***BMI [mean (SD)]*** | 24.1 (5.3) | 24.3 (5.3) | 23.9 (5.3) | 0.269 |
| ***Weight, kg [mean (SD)]*** | 68.8 (14.3) | 69.1 (14.5) | 68.4 (13.9) | 0.377 |
| ***eGFR [mean (SD)]*** | 115.9 (13.5) | 116.3 (14.2) | 115.4 (12.4) | 0.252 |
| ***CD4 count per uL [N (%)]*** |  |  |  | 0.529 |
| ≥ 300 | 514 (48.8) | 306 (50.1) | 208 (47.1) |  |
| 200 - 300 | 220 (20.9) | 121 (19.8) | 99 (22.4) |  |
| 100 - 200 | 196 (18.6) | 109 (17.8) | 87 (19.7) |  |
| < 100 | 123 (11.7) | 75 (12.3) | 48 (10.9) |  |
| ***Systolic BP, mmHg [mean (SD)]*** | 123 (15.4) | 124.6 (17.5) | 120.7 (11.5) | < 0.001 |
| ***Diastolic BP, mmHg [mean (SD)]*** | 79.2 (11.9) | 80.7 (13.3) | 77.1 (9.2) | < 0.001 |
| ***uACR, mg/mmol [mean (SD)]*** | 2.6 (11.4) | 3 (13) | 2.1 (8.6) | 0.169 |

sTable 6 legend: Results presented as N (%) or mean (SD). P-values estimated through either t-test or Chi-squared test, where appropriate. Abbreviations: BP = blood pressure, BMI = body mass index, CD4 = clusters of differentiation 4, DTG = Dolutegravir, eGFR = estimated glomerular filtration rate, FTC = Emtricitabine, kg = kilogram, mg = milligram, mmHG = millimeters of mercury, mmol = millimole, SD = standard deviation, TAF = Tenofovir Alafenamide, TDF = Tenofovir Disoproxil Fumarate, uACR = urine albumin-creatinine ratio, uL = microliter.

**sTable 7. Baseline characteristics of secondary analytical sample at 48 weeks by treatment group**

| **Characteristic** | **Overall**  **(N = 803)** | **TAF/FTC+DTG (N = 275)** | **TDF/FTC+DTG (N = 283)** | **TDF/FTC/EFV**  **(N = 245)** | **p-value** |
| --- | --- | --- | --- | --- | --- |
| ***Age [mean (SD)]*** | 31.9 (7.3) | 31.5 (7.4) | 31.4 (7.2) | 32 (7.1) | 0.889 |
| ***Sex [N (%)]*** |  |  |  |  | 0.410 |
| Female | 477 (59.4) | 172 (62.5) | 162 (57.2) | 143 (58.4) |  |
| Male | 326 (40.6) | 103 (37.5) | 121 (42.8) | 102 (41.6) |  |
| ***BMI [mean (SD)]*** | 23.8 (5.1) | 23.9 (5.0) | 23.7 (5.0) | 24 (5.7) | 0.765 |
| ***Weight, kg [mean (SD)]*** | 67.9 (13.7) | 67.6 (13.4) | 67.7 (13.2) | 68.6 (14.9) | 0.643 |
| ***eGFR [mean (SD)]*** | 117 (12.6) | 118.2 (12.1) | 117.6 (12.1) | 116 (13.4) | 0.281 |
| ***CD4 count per uL [N (%)]*** |  |  |  |  | 0.479 |
| ≥ 300 | 386 (36.7) | 141 (40.2) | 121 (34.5) | 124 (35.3) |  |
| 200 - 300 | 168 (16) | 55 (15.7) | 64 (18.2) | 49 (14) |  |
| 100 - 200 | 152 (14.4) | 46 (13.1) | 60 (17.1) | 46 (13.1) |  |
| < 100 | 97 (9.2) | 33 (9.4) | 38 (10.8) | 26 (7.4) |  |
| ***Systolic BP, mmHg [mean (SD)]*** | 120.3 (11.8) | 118.7 (11.6) | 120.2 (12.4) | 121.1 (12) | 0.323 |
| ***Diastolic BP, mmHg [mean (SD)]*** | 77 (9.4) | 76.5 (9.3) | 76.3 (10) | 77.8 (9.5) | 0.349 |
| ***uACR, mg/mmol [mean (SD)]*** | 2.6 (10.8) | 2 (6.4) | 3.3 (14.3) | 2.5 (10.5) | 0.546 |

sTable 7 legend: Results presented as N (%) or mean (SD). P-values estimated through either ANOVA or Chi-squared test, where appropriate. Abbreviations: BP = blood pressure, BMI = body mass index, CD4 = clusters of differentiation 4, DTG = Dolutegravir, eGFR = estimated glomerular filtration rate, FTC = Emtricitabine, kg = kilogram, mg = milligram, mmHG = millimeters of mercury, mmol = millimole, SD = standard deviation, TAF = Tenofovir Alafenamide, TDF = Tenofovir Disoproxil Fumarate, uACR = urine albumin-creatinine ratio, uL = microliter.

**sTable 8. Baseline characteristics of secondary analytical sample at 96 weeks by treatment group**

| **Characteristic** | **Overall**  **(N = 752)** | **TAF/FTC+DTG (N = 259)** | **TDF/FTC+DTG (N = 259)** | **TDF/FTC/EFV**  **(N = 234)** | **p-value** |
| --- | --- | --- | --- | --- | --- |
| ***Age [mean (SD)]*** | 32 (7.3) | 31.3 (7.3) | 31.5 (7.3) | 32 (7.3) | 0.637 |
| ***Sex [N (%)]*** |  |  |  |  | 0.325 |
| Female | 446 (59.3) | 163 (62.9) | 147 (56.8) | 136 (58.1) |  |
| Male | 306 (40.7) | 96 (37.1) | 112 (43.2) | 98 (41.9) |  |
| ***BMI [mean (SD)]*** | 23.8 (5.1) | 23.9 (5) | 23.6 (5.1) | 23.9 (5.5) | 0.894 |
| ***Weight, kg [mean (SD)]*** | 67.8 (13.5) | 67.2 (13.2) | 67.4 (13.2) | 68.6 (14.7) | 0.611 |
| ***eGFR [mean (SD)]*** | 116.7 (12.7) | 118.1 (12.1) | 117.5 (12.2) | 115.8 (13.3) | 0.448 |
| ***CD4 count per uL [N (%)]*** |  |  |  |  | 0.588 |
| ≥ 300 | 359 (34.1) | 130 (37) | 111 (31.6) | 118 (33.6) |  |
| 200 - 300 | 161 (15.3) | 52 (14.8) | 62 (17.7) | 47 (13.4) |  |
| 100 - 200 | 142 (13.5) | 44 (12.5) | 54 (15.4) | 44 (12.5) |  |
| < 100 | 90 (8.5) | 33 (9.4) | 32 (9.1) | 25 (7.1) |  |
| ***Systolic BP, mmHg [mean (SD)]*** | 120.6 (11.7) | 118.4 (11.4) | 120.4 (12.2) | 120.7 (11.4) | 0.258 |
| ***Diastolic BP, mmHg [mean (SD)]*** | 77.1 (9.3) | 76.3 (9.1) | 76.4 (10.0) | 77.4 (9.3) | 0.449 |
| ***uACR, mg/mmol [mean (SD)]*** | 2.6 (11.1) | 2.2 (6.8) | 3.2 (15.0) | 1.9 (6.3) | 0.730 |

sTable 8 legend: Results presented as N (%) or mean (SD). P-values estimated through either ANOVA or Chi-squared test, where appropriate. Abbreviations: BP = blood pressure, BMI = body mass index, CD4 = clusters of differentiation 4, DTG = Dolutegravir, eGFR = estimated glomerular filtration rate, FTC = Emtricitabine, kg = kilogram, mg = milligram, mmHG = millimeters of mercury, mmol = millimole, SD = standard deviation, TAF = Tenofovir Alafenamide, TDF = Tenofovir Disoproxil Fumarate, uACR = urine albumin-creatinine ratio, uL = microliter.

**sTable 9. Baseline characteristics of secondary analytical sample at 192 weeks by treatment group**

| **Characteristic** | **Overall**  **(N = 512)** | **TAF/FTC+DTG**  **(N = 192)** | **TDF/FTC+DTG**  **(N = 174)** | **TDF/FTC/EFV**  **(N = 146)** | **p-value** |
| --- | --- | --- | --- | --- | --- |
| ***Age [mean (SD)]*** | 32.9 (6.9) | 32.1 (6.6) | 32.6 (7.4) | 32.8 (6.9) | 0.941 |
| ***Sex [N (%)]*** |  |  |  |  | 0.210 |
| Female | 291 (56.8) | 118 (61.5) | 97 (55.7) | 76 (52.1) |  |
| Male | 221 (43.2) | 74 (38.5) | 77 (44.3) | 70 (47.9) |  |
| ***BMI [mean (SD)]*** | 23.9 (5.1) | 23.8 (4.8) | 23.8 (5.3) | 24.1 (5.8) | 0.892 |
| ***Weight, kg [mean (SD)]*** | 68.3 (13.5) | 67.7 (12.8) | 67.8 (13.4) | 69.8 (15.5) | 0.415 |
| ***eGFR [mean (SD)]*** | 114.8 (12.8) | 116 (11.9) | 116.3 (12.2) | 113.5 (13.1) | 0.408 |
| ***CD4 count per uL [N (%)]*** |  |  |  |  | 0.400 |
| ≥ 300 | 235 (22.3) | 94 (26.8) | 68 (19.4) | 73 (20.8) |  |
| 200 - 300 | 111 (10.5) | 37 (10.5) | 46 (13.1) | 28 (8) |  |
| 100 - 200 | 101 (9.6) | 37 (10.5) | 35 (10) | 29 (8.3) |  |
| < 100 | 65 (6.2) | 24 (6.8) | 25 (7.1) | 16 (4.6) |  |
| ***Systolic BP, mmHg [mean (SD)]*** | 121.7 (11.4) | 119.2 (10.2) | 121.9 (12.3) | 121.2 (11.9) | 0.424 |
| ***Diastolic BP, mmHg [mean (SD)]*** | 77.9 (9.2) | 76.6 (8.1) | 77 (9.9) | 77.8 (9.7) | 0.734 |
| ***uACR, mg/mmol [mean (SD)]*** | 2.4 (10.3) | 1.6 (3.6) | 2.7 (13.2) | 1.9 (6) | 0.738 |

sTable 9 legend: Results presented as N (%) or mean (SD). P-values estimated through either ANOVA or Chi-squared test, where appropriate. Abbreviations: BP = blood pressure, BMI = body mass index, CD4 = clusters of differentiation 4, DTG = Dolutegravir, eGFR = estimated glomerular filtration rate, FTC = Emtricitabine, kg = kilogram, mg = milligram, mmHG = millimeters of mercury, mmol = millimole, SD = standard deviation, TAF = Tenofovir Alafenamide, TDF = Tenofovir Disoproxil Fumarate, uACR = urine albumin-creatinine ratio, uL = microliter.

**sTable 10. Changes in systolic and diastolic blood pressure (mmHg) from baseline to weeks 48 and 96, by ADVANCE treatment groups**

|  | **48 weeks** | | **96 weeks** | |
| --- | --- | --- | --- | --- |
|  | **Δ Systolic** | **Δ Diastolic** | **Δ Systolic** | **Δ Diastolic** |
| **TAF/3TC/DTG** | 1.19 (-0.34 to 2.72) | 1.19 (-0.06 to 2.44) | 1.67 (-0.01 to 3.35) | 2.48 (1.18 to 3.79) |
| **TDF/3TC/DTG** | -0.41 (-2.07 to 1.26) | 0.88 (-0.44 to 2.20) | -0.48 (-2.22 to 1.26) | 1.00 (-0.35 to 2.34) |
| **TDF/3TC/EFV** | -2.47 (-4.05 to -0.88) | -0.54 (-1.79 to 0.71) | -2.10 (-3.82 to -0.37) | -0.49 (-1.79 to 0.82) |

sTable 10 legend: Estimates of the mean change in blood pressure measures, with 95% confidence intervals. Sample restricted to those participants who did not report use of hypertension medication at baseline or up to week 48 or 96 when applicable, and had non-missing measures of systolic or diastolic blood pressure. Abbreviations: DTG = Dolutegravir, FTC = Emtricitabine, mmHG = millimeters of mercury, TAF = Tenofovir Alafenamide, TDF = Tenofovir Disoproxil Fumarate

**sTable 11. Changes in systolic and diastolic blood pressure (mmHg) from baseline to week 192, by ADVANCE treatment groups**

|  | **192 weeks** | |
| --- | --- | --- |
|  | **Δ Systolic** | **Δ Diastolic** |
| **TAF/3TC/DTG** | 3.56 (1.67 to 5.44) | 4.81 (3.34 to 6.27) |
| **TDF/3TC/DTG** | 0.65 (-1.42 to 2.71) | 2.63 (0.96 to 4.3) |
| **TDF/3TC/EFV** | -0.40 (-2.21 to 1.42) | 1.67 (0.09 to 3.26) |

sTable 11 legend: Estimates of the mean change in blood pressure measures, with 95% confidence intervals. Sample restricted to those participants who did not report use of hypertension medication at baseline or up to week 192 when, and had non-missing measures of systolic or diastolic blood pressure. Abbreviations: DTG = Dolutegravir, FTC = Emtricitabine, mmHG = millimeters of mercury, TAF = Tenofovir Alafenamide, TDF = Tenofovir Disoproxil Fumarate

**sTable** **12. Proportion with treatment emergent hypertension and grade at weeks 48, 96, and 192, by ADVANCE treatment group**

1. **48 weeks, n = 803**

|  | **Grade 1** | **Grade 2** | **Grade 3** | **Total** |
| --- | --- | --- | --- | --- |
| **DTG/TAF/FTC** | 14 (5.1) | 14 (5.1) | 5 (1.8) | 33 (12) |
| **DTG/TDF/FTC** | 19 (6.7) | 12 (4.2) | 5 (1.8) | 36 (12.7) |
| **EFV/TDF/FTC** | 12 (4.9) | 3 (1.2) | 4 (1.6) | 19 (7.8) |

1. **96 weeks, n = 752**

|  | **Grade 1** | **Grade 2** | **Grade 3** | **Total** |
| --- | --- | --- | --- | --- |
| **DTG/TAF/FTC** | 15 (5.8) | 26 (10.0) | 6 (2.3) | 47 (18.1) |
| **DTG/TDF/FTC** | 18 (6.9) | 17 (6.6) | 5 (1.9) | 40 (15.4) |
| **EFV/TDF/FTC** | 13 (5.6) | 14 (6.0) | 4 (1.7) | 31 (13.2) |

1. **192 weeks, n = 512**

|  | **Grade 1** | **Grade 2** | **Grade 3** | **Total** |
| --- | --- | --- | --- | --- |
| **DTG/TAF/FTC** | 20 (10.4) | 23 (12) | 6 (3.1) | 49 (25.5) |
| **DTG/TDF/FTC** | 17 (9.8) | 14 (8) | 4 (2.3) | 35 (20.1) |
| **EFV/TDF/FTC** | 12 (8.2) | 10 (6.8) | 4 (2.7) | 26 (17.8) |

sTable 12 legend: Estimates of the proportion of sample at each time point that have been deemed to have grade 1 hypertension, grade 2 hypertension, grade 3 hypertension, and any hypertension by study standards. Results displayed as N (%). Sample restricted to those participants who did not report use of hypertension medication at baseline or had two hypertensive blood pressure measures at baseline, and had non-missing blood pressure measures at 48, 96, or 192 weeks. Abbreviations: DTG = Dolutegravir, FTC = Emtricitabine, TAF = Tenofovir Alafenamide, TDF = Tenofovir Disoproxil Fumarate

**sTable 13. Linear regression models of the association of individual-level characteristics and change in systolic blood pressure (mmHg) from baseline to week 48, adjusted and unadjusted.**

|  | **Δ SBP at 48 weeks** | |
| --- | --- | --- |
| **Characteristic** | **Unadjusted** | **Adjusted** |
| ***Age*** | -0.01 (-0.13 to 0.12) | -0.01 (-0.14 to 0.11) |
| ***Sex*** |  |  |
| Female | 1.94 (0.05 to 3.82) | 1.36 (-0.53 to 3.26) |
| ***Group*** |  |  |
| TDF/FTC+DTG | 2.06 (-0.21 to 4.32) | 1.74 (-0.76 to 4.24) |
| TAF/FTC+DTG | 3.65 (1.38 to 5.93) | 2.42 (-0.12 to 4.97) |
| ***Change in BMI*** | 1.10 (0.68 to 1.52) | 0.86 (0.41 to 1.32) |
| ***Change in eGFR*** | -0.04 (-0.11 to 0.02) | 0.01 (-0.06 to 0.09) |
| ***CD4, count per ul*** |  |  |
| 200 - 300 | -1.77 (-4.18 to 0.63) | -1.86 (-4.26 to 0.53) |
| 100 - 200 | 1.62 (-0.87 to 4.12) | 1.19 (-1.35 to 3.73) |
| < 100 | 3.03 (0.04 to 6.01) | 1.82 (-1.26 to 4.89) |
| ***N observations*** |  | 765 |

sTable 13 legend: Results are presented as point estimates (95% CI). Estimates derived from ordinary least-squares linear regression models. Unadjusted models are univariable models in which change in systolic blood pressure is regressed on characteristic. Adjusted models are multivariable models in which change in systolic blood pressure is regressed on all of the included characteristics. ‘Change in BMI’ is the difference between baseline BMI and BMI at week 48, measured in kg/m^2^. ‘Change in eGFR’ is the difference between baseline eGFR and eGFR at week 48, measured in mL/minutes/1.73 m^2^. CD4 measured at baseline. Sample restricted to those participants who did not report use of hypertension medication at baseline or up to week 48, and had non-missing measures of systolic or diastolic blood pressure. Abbreviations: BMI = body mass index, CD4= clusters of differentiation 4, DTG = Dolutegravir, eGFR = estimated glomerular filtration rate, FTC = Emtricitabine, mmHG = millimeters of mercury, SBP = systolic blood pressure, TAF = Tenofovir Alafenamide, TDF = Tenofovir Disoproxil Fumarate, uL = microliter.

**sTable 14. Linear regression models of the association of individual-level characteristics and change in diastolic blood pressure (mmHg) from baseline, adjusted and unadjusted.**

|  | **Δ DBP at 96 weeks** | | **Δ DBP at 192 weeks** | |
| --- | --- | --- | --- | --- |
| **Characteristic** | **Unadjusted** | **Adjusted** | **Unadjusted** | **Adjusted** |
| ***Age*** | -0.09 (-0.19 to 0.02) | -0.08 (-0.19 to 0.02) | 0.04 (-0.10 to 0.17) | 0.03 (-0.11 to 0.16) |
| ***Sex*** |  |  |  |  |
| Female | 1.35 (-0.20 to 2.91) | 0.74 (-0.85 to 2.33) | -0.10 (-1.98 to 1.79) | -0.62 (-2.58 to 1.34) |
| ***Group*** |  |  |  |  |
| TAF/FTC+DTG | 2.97 (1.10 to 4.85) | 2.12 (0.01 to 4.24) | 2.37 (0.06 to 4.67) | 0.70 (-1.83 to 3.23) |
| TDF/FTC+DTG | 1.48 (-.38 to 3.35) | 1.28 (-0.81 to 3.37) | 1.06 (-1.27 to 3.38) | -0.19 (-2.72 to 2.33) |
| ***Change in BMI*** | 0.69 (0.40 to 0.98) | 0.52 (0.20 to 0.84) | 0.59 (0.26 to 0.93) | 0.49 (0.12 to 0.87) |
| ***Change in eGFR*** | -0.03 (-0.09 to 0.02) | 0.02 (-0.05 to 0.08) | -0.09 (-0.15 to -0.02) | -0.06 (-0.13 to 0.01) |
| ***CD4, count per ul*** |  |  |  |  |
| 200 - 300 | -1.40 (-3.36 to 0.56) | -1.47 (-3.43 to 0.49) | -1.31 (-3.70 to 1.08) | -1.78 (-4.18 to 0.63) |
| 100 - 200 | 1.35 (-0.71 to 3.40) | 1.13 (-0.98 to 3.24) | 2.23 (-0.26 to 4.73) | 1.12 (-1.48 to 3.71) |
| < 100 | 2.73 (0.22 to 5.23) | 1.94 (-0.66 to 4.54) | 2.20 (-0.93 to 5.33) | 1.07 (-2.15 to 4.29) |
| ***N observations*** |  | 686 |  | 442 |

Table 14 legend: Results are presented as point estimates (95% CI). Estimates derived from ordinary least-squares linear regression models. Unadjusted models are univariable models in which change in diastolic blood pressure is regressed on characteristic. Adjusted models are multivariable models in which change in diastolic blood pressure is regressed on all of the included characteristics. ‘Change in BMI’ is the difference between baseline BMI and BMI at week 96 and 192, respectively, measured in kg/m^2^. ‘Change in eGFR’ is the difference between baseline eGFR and eGFR at week 96 and 192, respectively, measured in mL/minutes/1.73 m^2^. CD4 measured at baseline. Sample restricted to those participants who did not report use of hypertension medication at baseline or up to week 96 or 192 when applicable, and had non-missing measures of systolic or diastolic blood pressure. Abbreviations: BMI = body mass index, CD4= clusters of differentiation 4, DBP = diastolic blood pressure, DTG = Dolutegravir, eGFR = estimated glomerular filtration rate, FTC = Emtricitabine, mmHG = millimeters of mercury, TAF = Tenofovir Alafenamide, TDF = Tenofovir Disoproxil Fumarate, uL = microliter.

**sTable 15. Linear regression models of the association of individual-level characteristics and change in diastolic blood pressure (mmHg) from baseline to week 48, adjusted and unadjusted.**

|  | **Δ DBP at 48 weeks** | |
| --- | --- | --- |
| **Characteristic** | **Unadjusted** | **Adjusted** |
| ***Age*** | -0.03 (-0.13 to 0.08) | -0.02 (-0.12 to 0.08) |
| ***Sex*** |  |  |
| Female | 0.10 (-1.40 to 1.60) | -0.25 (-1.78 to 1.28) |
| ***Group*** |  |  |
| TAF/FTC+DTG | 1.73 (-0.09 to 3.54) | 0.83 (-1.22 to 2.89) |
| TDF/FTC+DTG | 1.42 (-0.39 to 3.22) | 0.88 (-1.14 to 2.90) |
| ***Change in BMI*** | 0.42 (0.08 to 0.75) | 0.41 (0.04 to 0.78) |
| ***Change in eGFR*** | -0.04 (-0.09 to 0.01) | -0.02 (-0.08 to 0.04) |
| ***CD4, count per ul*** |  |  |
| 200 - 300 | -1.77 (-3.69 to 0.15) | -1.90 (-3.84 to 0.04) |
| 100 - 200 | 1.06 (-0.93 to 3.05) | 0.66 (-1.39 to 2.70) |
| < 100 | -1.03 (-3.41 to 1.34) | -1.76 (-4.24 to 0.72) |
| ***N observations*** |  | 764 |

sTable 15 legend: Results are presented as point estimates (95% CI). Estimates derived from ordinary least-squares linear regression models. Unadjusted models are univariable models in which change in systolic blood pressure is regressed on characteristic. Adjusted models are multivariable models in which change in systolic blood pressure is regressed on all of the included characteristics. ‘Change in BMI’ is the difference between baseline BMI and BMI at week 48, measured in kg/m^2^. ‘Change in eGFR’ is the difference between baseline eGFR and eGFR at week 48, measured in mL/minutes/1.73 m^2^. CD4 measured at baseline. Sample restricted to those participants who did not report use of hypertension medication at baseline or up to week 48, and had non-missing measures of systolic or diastolic blood pressure. Abbreviations: BMI = body mass index, CD4= clusters of differentiation 4, DBP = diastolic blood pressure, DTG = Dolutegravir, eGFR = estimated glomerular filtration rate, FTC = Emtricitabine, mmHG = millimeters of mercury, TAF = Tenofovir Alafenamide, TDF = Tenofovir Disoproxil Fumarate, uL = microliter.

**sTable 16. Poisson regression models of the association of individual-level characteristics and risk of emergent-hypertension up until week 48, adjusted and unadjusted.**

|  | **RR (95% CI) at 48 weeks** | |
| --- | --- | --- |
| **Characteristic** | **Unadjusted** | **Adjusted** |
| ***Age*** | 1.07 (1.05 to 1.09) | 1.07 (1.05 to 1.09) |
| ***Sex*** |  |  |
| Female | 0.78 (0.53 to 1.16) | 0.86 (0.58 to 1.28) |
| ***Group*** |  |  |
| DTG/TAF/FTC | 1.55 (0.90 to 2.65) | 1.74 (0.94 to 3.21) |
| DTG/TDF/FTC | 1.64 (0.97 to 2.78) | 1.83 (1.00 to 3.37) |
| ***Change in BMI*** | 1.01 (0.92 to 1.10) | 0.98 (0.89 to 1.08) |
| ***Change in eGFR*** | 1.00 (0.99 to 1.01) | 1.01 (0.99 to 1.02) |
| ***CD4, count per ul*** |  |  |
| 200 - 300 | 0.91 (0.53 to 1.55) | 0.80 (0.48 to 1.35) |
| 100 - 200 | 0.89 (0.51 to 1.55) | 0.78 (0.45 to 1.37) |
| < 100 | 1.20 (0.67 to 2.15) | 0.97 (0.52 to 1.78) |
| ***N observations*** |  | 803 |

sTable 16 legend: Results are presented as risk ratios (95% CI). Estimates derived from Poisson regression models with robust standard errors. Unadjusted models are univariable models in which treatment emergent hypertension is regressed on characteristic. Adjusted models are multivariable models in which treatment emergent hypertension is regressed on all of the included characteristics. ‘Change in BMI’ is the difference between baseline BMI and BMI at week 48, measured in kg/m^2^. ‘Change in eGFR’ is the difference between baseline eGFR and eGFR at week 48, measured in mL/minutes/1.73 m^2^. CD4 measured at baseline. Sample restricted to those participants who did not report use of hypertension medication at baseline or had two hypertensive blood pressure measures at baseline. Abbreviations: BMI = body mass index, CD4= clusters of differentiation 4, DTG = Dolutegravir, eGFR = estimated glomerular filtration rate, FTC = Emtricitabine, RR = risk ratio, SBP = systolic blood pressure, TAF = Tenofovir Alafenamide, TDF = Tenofovir Disoproxil Fumarate, uL = microliter.

**sTable 17. Sex-stratified linear regression models of the association of individual-level characteristics and change in systolic blood pressure (mmHg) from baseline to week 48, adjusted and unadjusted.**

|  | **Male** | | **Female** | |
| --- | --- | --- | --- | --- |
| **Characteristic** | **Unadjusted** | **Adjusted** | **Unadjusted** | **Adjusted** |
| ***Age*** | -.03 (-.23 to .17) | -0.06 (-0.26 to .013) | 0.03 (-0.14 to 0.19) | 0.01 (-0.16 to 0.18) |
| ***Group*** |  |  |  |  |
| TAF/FTC+DTG | 4.46 (0.93 to 8.00) | 2.42 (-1.51 to 6.35) | 2.99 (0.01 to 5.98) | 2.2 (-1.18 to 5.57) |
| TDF/FTC+DTG | 2.15 (-1.19 to 5.49) | 0.94 (-2.79 to 4.66) | 2.08 (-0.98 to 5.15) | 2.07 (-1.32 to 5.47) |
| ***Change in BMI*** | 1.77 (0.97 to 2.57) | 1.56 (0.69 to 2.43) | 0.82 (0.32 to 1.32) | 0.67 (0.12 to 1.22) |
| ***Change in eGFR*** | -0.07 (-0.17 to .03) | -0.01 (-0.13 to 0.10) | -0.02 (-0.11 to 0.06) | 0.03 (-0.07 to 0.12) |
| ***CD4, count per ul*** |  |  |  |  |
| 200 - 300 | -3.93 (-7.58 to -0.28) | -4.14 (-7.77 to -0.52) | -0.06 (-3.26 to 3.13) | -0.35 (-3.56 to 2.86) |
| 100 - 200 | 1.27 (-2.38 to 4.93) | 0.53 (-3.17 to 4.23) | 2.17 (-1.25 to 5.60) | 1.39 (-2.11 to 4.89) |
| < 100 | 3.10 (-1.19 to 7.40) | 0.93 (-3.49 to 5.36) | 3.2 (-0.93 to 7.34) | 1.86 (-2.43 to 6.16) |
| ***N observations*** |  | 309 |  | 456 |

sTable 17 legend: Results are presented as point estimates (95% CI). Estimates derived from ordinary least-squares linear regression models. Unadjusted models are univariable models in which change in systolic blood pressure is regressed on characteristic. Adjusted models are multivariable models in which change in systolic blood pressure is regressed on all of the included characteristics. ‘Change in BMI’ is the difference between baseline BMI and BMI at week 48, measured in kg/m^2^. ‘Change in eGFR’ is the difference between baseline eGFR and eGFR at week 48, measured in mL/minutes/1.73 m^2^. CD4 measured at baseline. Sample restricted to those participants who did not report use of hypertension medication at baseline or up to week 48, and had non-missing measures of systolic or diastolic blood pressure. Abbreviations: BMI = body mass index, CD4= clusters of differentiation 4, DTG = Dolutegravir, eGFR = estimated glomerular filtration rate, FTC = Emtricitabine, mmHG = millimeters of mercury, SBP = systolic blood pressure, TAF = Tenofovir Alafenamide, TDF = Tenofovir Disoproxil Fumarate, uL = microliter.

**sTable 18. Sex-stratified Poisson regression models of the association of individual-level characteristics and risk of emergent-hypertension up until week 48, adjusted and unadjusted.**

|  | **Male** | | **Female** | |
| --- | --- | --- | --- | --- |
| **Characteristic** | **Unadjusted** | **Adjusted** | **Unadjusted** | **Adjusted** |
| ***Age*** | 1.05 (1.02 to 1.09) | 1.05 (1.02 to 1.09) | 1.08 (1.05 to 1.1) | 1.08 (1.05 to 1.11) |
| ***Group*** |  |  |  |  |
| TAF/FTC+DTG | 2.83 (1.25 to 6.41) | 3.12 (1.23 to 7.94) | 0.90 (0.42 to 1.91) | 0.99 (0.43 to 2.29) |
| TDF/FTC+DTG | 1.69 (0.71 to 4.02) | 1.84 (0.66 to 5.15) | 1.62 (0.83 to 3.15) | 1.87 (0.90 to 3.88) |
| ***Change in BMI*** | 1.08 (0.91 to 1.27) | 0.96 (0.77 to 1.19) | 0.99 (0.89 to 1.11) | 1.02 (0.91 to 1.14) |
| ***Change in eGFR*** | 1.00 (0.98 to 1.02) | 1.01 (0.98 to 1.03) | 1.00 (0.98 to 1.02) | 1.01 (0.98 to 1.03) |
| ***CD4, count per ul*** |  |  |  |  |
| 200 - 300 | 0.54 (0.21 to 1.39) | 0.55 (0.22 to 1.41) | 1.22 (.64 to 2.34) | 1.00 (0.54 to 1.85) |
| 100 - 200 | 1.05 (0.51 to 2.15) | 1.14 (0.54 to 2.41) | 0.63 (0.25 to 1.58) | 0.53 (0.21 to 1.32) |
| < 100 | 1.26 (0.59 to 2.71) | 1.16 (0.49 to 2.74) | 1.02 (0.41 to 2.54) | 0.82 (0.31 to 2.12) |
| ***N observations*** |  | 326 |  | 477 |

sTable 18 legend: Results are presented as risk ratios (95% CI). Estimates derived from Poisson regression models with robust standard errors. Unadjusted models are univariable models in which baseline hypertension is regressed on characteristic. Adjusted models are multivariable models in which baseline hypertension is regressed on all of the included characteristics. Baseline hypertension defined as reported use of hypertension medication at baseline or two hypertensive blood pressure measures at baseline. Abbreviations: BMI = body mass index, CD4= clusters of differentiation 4, DTG = Dolutegravir, eGFR = estimated glomerular filtration rate, FTC = Emtricitabine, mmHG = millimeters of mercury, HTN= hypertension, TAF = Tenofovir Alafenamide, TDF = Tenofovir Disoproxil Fumarate, uL = microliter.

**sTable 19. Poisson regression models of the association of individual-level characteristics and risk of baseline hypertension, adjusted and unadjusted.**

|  | **RR (95% CI) at baseline** | |
| --- | --- | --- |
| **Characteristic** | **Unadjusted** | **Adjusted** |
| ***Age*** | 1.11 (1.09 to 1.12) | 1.10 (1.08 to 1.12) |
| ***Sex*** |  |  |
| Female | 0.80 (0.56 to 1.13) | 0.58 (0.39 to 0.86) |
| ***Group*** |  |  |
| TAF/FTC+DTG | 0.79 (0.52 to 1.21) | 0.82 (0.55 to 1.22) |
| TDF/FTC+DTG | 0.81 (0.53 to 1.24) | 0.75 (0.5 to 1.11) |
| ***BMI, kg/m^2^*** | 1.07 (1.05 to 1.09) | 1.08 (1.04 to 1.11) |
| ***eGFR*** | 0.96 (0.96 to 0.97) | 0.99 (0.98 to 1.01) |
| ***CD4, count per uL*** |  |  |
| 200 - 300 | 1.37 (0.90 to 2.10) | 1.09 (0.74 to 1.60) |
| 100 - 200 | 1.13 (0.71 to 1.81) | 0.98 (0.63 to 1.54) |
| < 100 | 0.74 (0.37 to 1.46) | 0.62 (0.33 to 1.16) |
| ***N observations*** |  | 1053 |

sTable 19 legend: Results are presented as risk ratios (95% CI). Estimates derived from Poisson regression models with robust standard errors. Unadjusted models are univariable models in which baseline hypertension is regressed on characteristic. Adjusted models are multivariable models in which baseline hypertension is regressed on all of the included characteristics. Baseline hypertension defined as reported use of hypertension medication at baseline or two hypertensive blood pressure measures at baseline. Abbreviations: BMI = body mass index, CD4= clusters of differentiation 4, DTG = Dolutegravir, eGFR = estimated glomerular filtration rate, FTC = Emtricitabine, mmHG = millimeters of mercury, HTN= hypertension, TAF = Tenofovir Alafenamide, TDF = Tenofovir Disoproxil Fumarate, uL = microliter.

**sFigure 1. Kaplan-Meier curves of time until hypertension incidence, by treatment group**


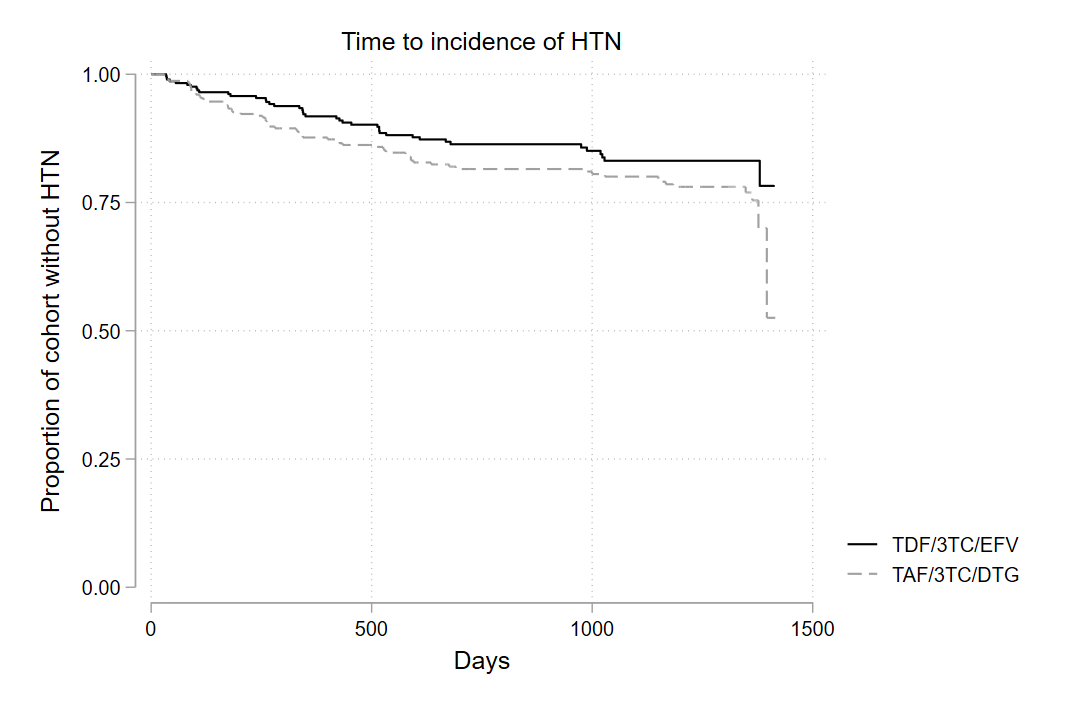


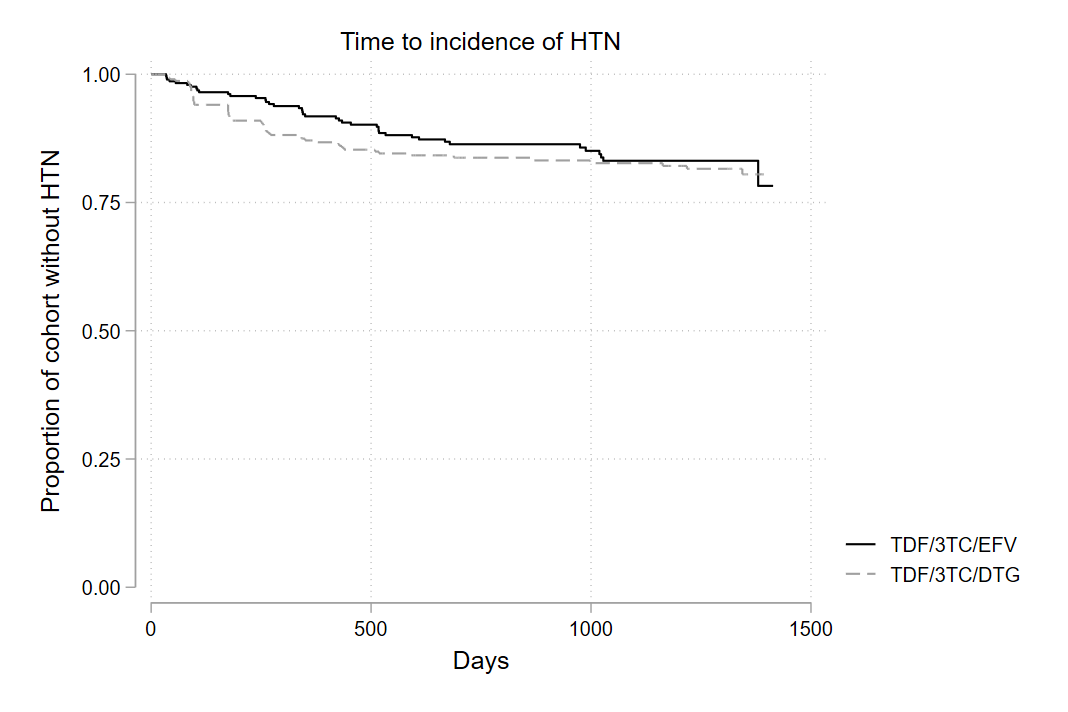


sFigure 1: Abbreviations: DTG = Dolutegravir, eGFR = estimated glomerular filtration rate, FTC = Emtricitabine, HTN= hypertension, TAF = Tenofovir Alafenamide, TDF = Tenofovir Disoproxil Fumarate

**sTable 20. Adjusted Cox-hazard model of time until hypertension diagnosis**

| **Characteristic** | **HR (95% CI)** |
| --- | --- |
| ***Age*** | 1.06 (1.04 to 1.09) |
| ***Female*** | 0.97 (0.69 to 1.37) |
| ***Group*** |  |
| DTG/TAF/FTC | 1.48 (0.99 to 2.20) |
| DTG/TDF/FTC | 1.21 (0.80 to 1.83) |
| ***Change in eGFR*** | 0.99 (0.98 to 1.00) |
| ***CD4 count per uL*** | |
| 200 - 300 | 0.99 (0.64 to 1.53) |
| 100 - 200 | 1.08 (0.70 to 1.67) |
| < 100 | 1.68 (1.09 to 2.59) |
| ***BMI*** |  |
| Normal | 1.00 (1.00 to 1.00) |
| Overweight | 1.00 (1.00 to 1.00) |
| Obese | 1.00 (1.00 to 1.00) |

sTable 20: Results presented as hazard ratios with 95% confidence intervals. Hypertension event determined by the visit at which there is a second hypertensive measure recorded and/ or started on antihypertensive medication. BMI category treated as time-varying covariate. Abbreviations: BMI = body mass index, CD4= clusters of differentiation 4, DTG = Dolutegravir, eGFR = estimated glomerular filtration rate, FTC = Emtricitabine, mmHG = millimeters of mercury, HTN= hypertension, TAF = Tenofovir Alafenamide, TDF = Tenofovir Disoproxil Fumarate, uL = microliter.

**sTable 21. Urine albumin:creatinine change and SBP change at 48 and 96 weeks by group**

| Group | Week of Follow-up | Spearman Correlation Coefficient | p |
| --- | --- | --- | --- |
| TDF/FTC/EFV | 48 | -0.0632 | 0.3292 |
| TDF/FTC+DTG | 48 | 0.0754 | 0.2218 |
| TAF/FTC+DTG | 48 | 0.0849 | 0.1741 |
| TDF/FTC/EFV | 96 | -0.0179 | 0.7918 |
| TDF/FTC+DTG | 96 | 0.0133 | 0.8408 |
| TAF/FTC+DTG | 96 | 0.0672 | 0.3094 |
